# Supplementary material for: N-Cadherin mRNA Levels in Peripheral Blood Could Be a Potential Indicator of New Metastases in Breast Cancer: A Pilot Study
Source: Int J Mol Sci. 2020 Jan 14;21(2):511. doi: 10.3390/ijms21020511 (PMC7013704; doi:10.3390/ijms21020511)
Supplement: Supplementary file 1 [file ijms-21-00511-s001.zip › Supplementary Materials/Masuda T. Table S1.docx]

| **Table S1. The patients for the expression analyses in PB.** | | | | | |
| --- | --- | --- | --- | --- | --- |
| no | drug | subtype | site of relapse | pretreatment drugs  after relapse | style of disease progression |
| 1 | S-1 | ER+HER2- | bone | PTX^b^+BV ^c^ | - |
| 2 | S-1 | ER+HER2- | lung, bone | HR^d^ | - |
| 3 | S-1 | ER+HER2- | liver, bone, lymph nodes | - | NM^g^+PEM^h^ |
| 4 | S-1 | ER+HER2- | lung, bone | HR、PTX+BV、HR | - |
| 5 | S-1 | TN^a^ | liver, bone | - | NM+PEM |
| 6 | S-1 | ER+HER2- | liver, bone, lymph nodes | HR | - |
| 7 | S-1 | TN | lymph nodes | - | PEM |
| 8 | S-1 | ER+HER2- | liver, bone, lymph nodes | HR | NM |
| 9 | Eribulin | TN | lung | Cape ^e^ | PEM |
| 10 | Eribulin | TN, ER+HER2- | pleura, lymph nodes | PTX+BV、S-1 | NM+PEM |
| 11 | Eribulin | ER+HER2- | lymph nodes | HR、PTX+BV、VNB^f^ | PEM |
| 12 | Eribulin | ER+HER2- | lung | PTX+BV | - |
| ^a^TN, Triple negative ^b^PTX, Paclitaxel ^c^BV, Bevacizumab ^d^HR, Hormone ^e^Cape, Capecitabine ^f^VNB, Vinorelbine ^g^NM, new metastasis ^h^PEM, pre-existing metastasis | | | | | |
|  |  |  |  |  |  |
