# Supplementary figures and images for: N-Cadherin mRNA Levels in Peripheral Blood Could Be a Potential Indicator of New Metastases in Breast Cancer: A Pilot Study
Source: Int J Mol Sci. 2020 Jan 14;21(2):511. doi: 10.3390/ijms21020511 (PMC7013704; doi:10.3390/ijms21020511)

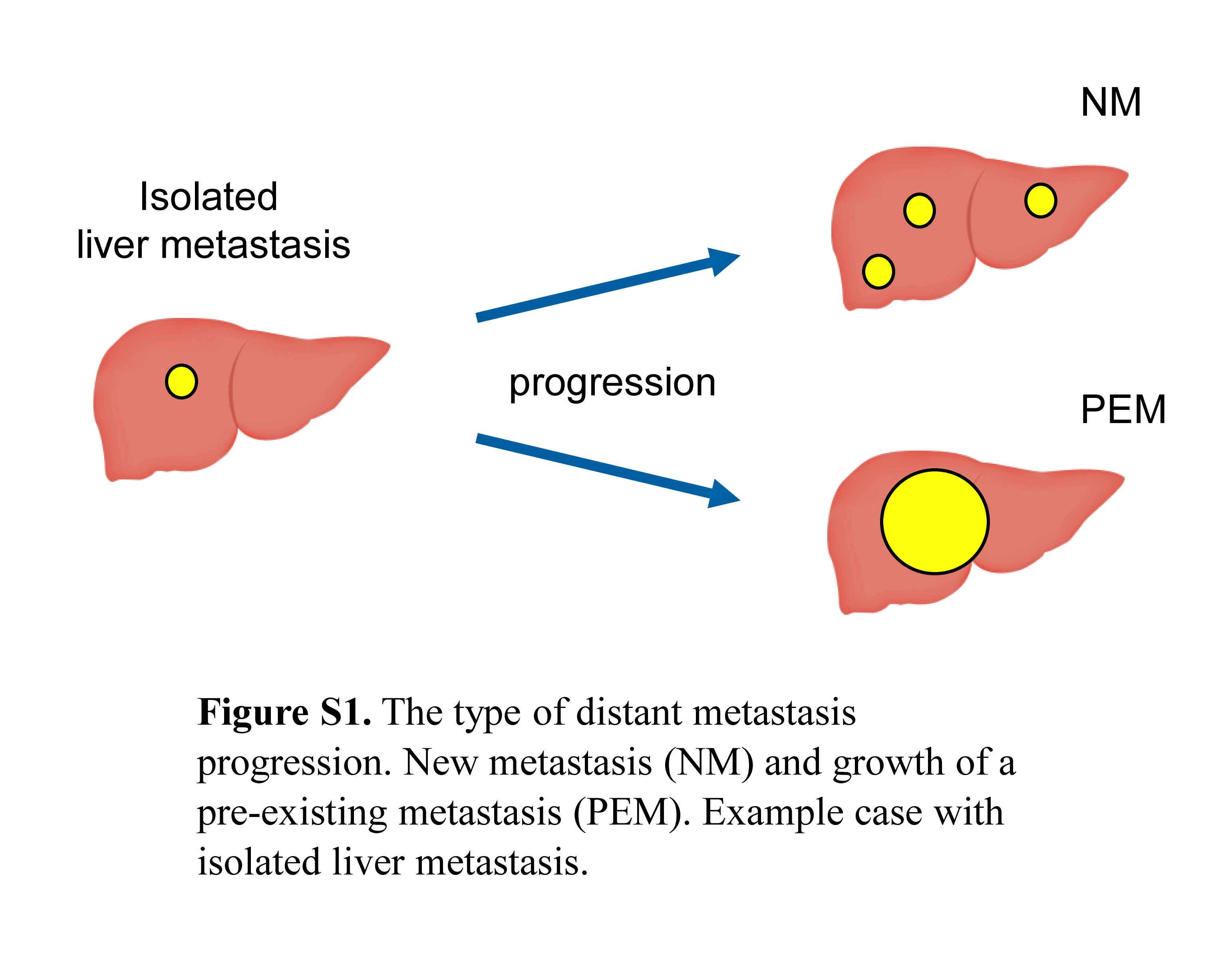

Supplement: Supplementary file 1 [file ijms-21-00511-s001.zip › Supplementary Materials/Masuda T. Figure S1.tif]

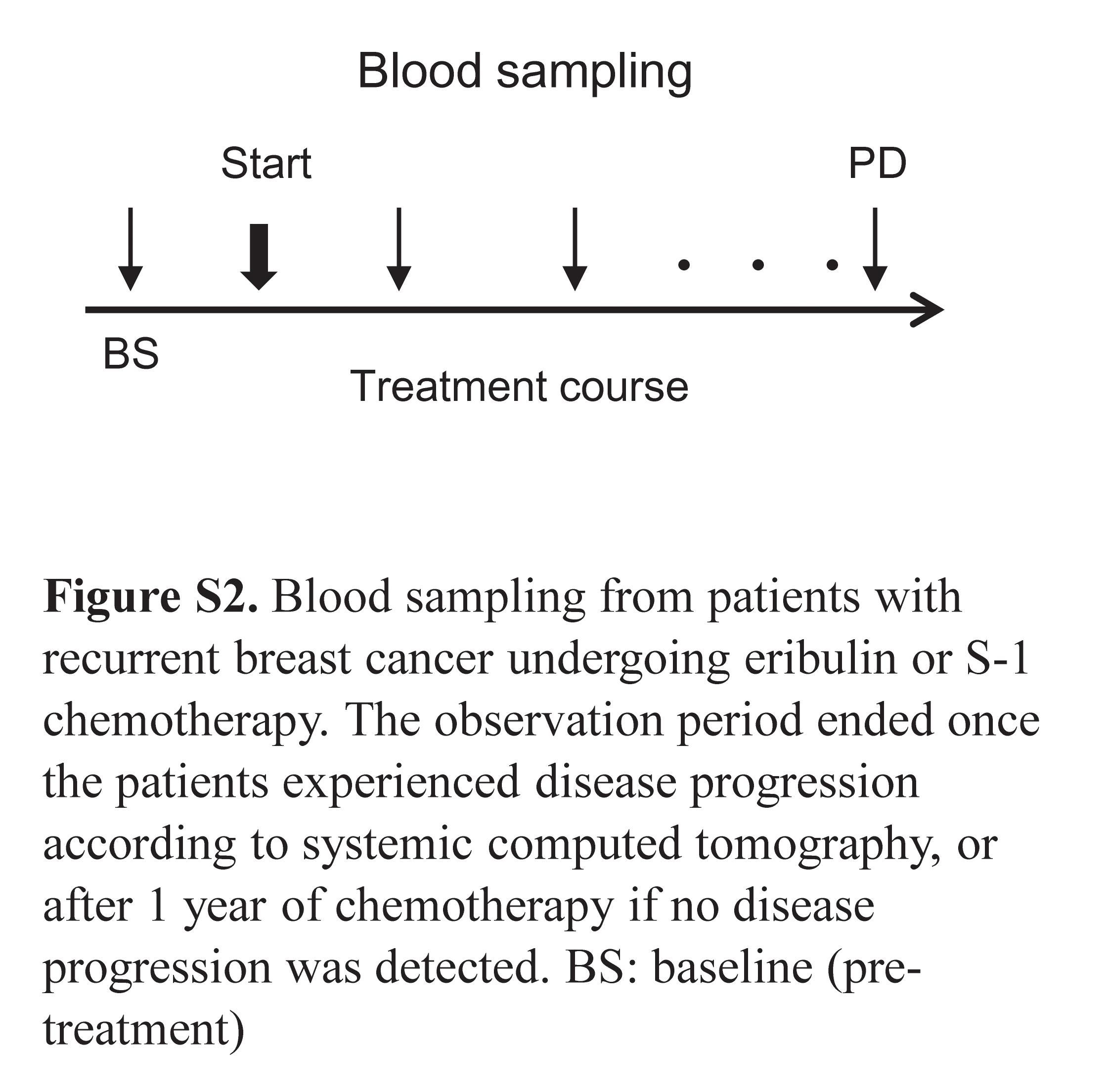

Supplement: Supplementary file 1 [file ijms-21-00511-s001.zip › Supplementary Materials/revised Figure S2.tif]
